# Does leisure activity matter for epigenetic aging? Analyses of arts engagement and physical activity in the UK Household Longitudinal Study

**DOI:** 10.1093/geroni/igag038

**Published:** 2026-05-11

**Authors:** Daisy Fancourt, Lehané Masebo, Saoirse Finn, Hei Wan Mak, Feifei Bu

**Affiliations:** Department of Behavioural Science and Health, University College London, London, United Kingdom; Division of Psychiatry, University College London, London, United Kingdom; Department of Behavioural Science and Health, University College London, London, United Kingdom; Department of Behavioural Science and Health, University College London, London, United Kingdom; Department of Behavioural Science and Health, University College London, London, United Kingdom

**Keywords:** Epigenetic aging, Lifestyle, Arts and cultural engagement, Physical activities

## Abstract

**Background and Objectives:**

Over the past decade, aging clocks have become widely adopted as important tools for understanding biological aging and have been redefining notions of “pro-longevity” lifestyles. However, this work is still at an early stage. Some leisure activities, such as arts and cultural engagement (ACEng), have never been studied at all, while others, such as physical activity (PA), have only received scant attention.

**Research Design and Methods:**

This study used data from 3,556 adults (2010–2012) in the UK Household Longitudinal Study, a large, nationally-representative cohort study, which includes 7 derived epigenetic clocks. We used a doubly robust estimation using the inverse-probability-weighted regression adjustment estimator adjusted for demographic, socioeconomic, data collection gaps, and technical covariates of epigenetic clocks.

**Results:**

ACEng and PA were related to slower epigenetic aging in the PhenoAge, DunedinPoAm and DunedinPACE clocks, although not to the other measured clocks (Lin, Horvath 2018, Horvath 2013, and Hannum), with comparable effect sizes between ACEng and PA. Evidence was consistently found across different measures of engagement, including diversity and frequency for ACEng, as well as frequency, diversity, and activeness for PA. These results were generally stronger amongst middle-aged and older adults aged 40 or above.

**Discussion and Implications:**

Our study provides the first evidence that ACEng, a much more recently recognized health behavior, is related to epigenetic aging, with magnitudes comparable to PA. These findings position ACEng as a potential contributor to healthy aging at the biological level, supporting its inclusion in public health strategies.

Innovation and Translational Significance: This study provides the first evidence that engaging in arts and cultural activities is associated with a slower pace of biological aging, with benefits similar to physical activity. Based on high-quality data and robust methods, these findings extend the existing literature on arts and health, positioning arts and cultural engagement as a novel, modifiable factor for healthy aging at the biological level. The evidence provides a scientific basis for integrating arts and cultural activities into public health frameworks and individual health behaviors, presenting an accessible and enriching pathway to healthy aging.

With aging populations becoming a global phenomenon, how to support not just a longer lifespan but also an increased “healthspan” is becoming a key question for both individuals and health services (The Lancet Public Health, 2017). Helping adults stay free from disease, maintain functional independence, and reduce the need for health services are key priorities for governments internationally (Beard & Bloom, 2015). Over the past two decades, theoretical and technological advances within molecular biology have identified a series of fundamental biological hallmarks of aging, including various molecular, cellular, and systemic processes underpinning health and disease (López-Otín et al., 2023). One of these is epigenetic alterations, including alterations in DNA methylation (DNAm) patterns, aberrant chromatin remodeling, abnormal post-translational modification of histones, and deregulated function of non-coding RNAs. Environmental stress accumulated over the lifetime disrupts epigenetic profiles, and this contributes to the aging phenotype by promoting instability, carcinogenesis, and cardiovascular pathologies (Pagiatakis et al., 2021; Zhang et al., 2020). In recent years, there have been major developments in biohorology: the use of aging “clocks” that are built from mapping patterns of DNAm across sparse but informative sets of specific CpG (cytosine-phospho-guanine) sites on the genome (Horvath & Raj, 2018). These epigenetic clocks are used to identify discrepancies between chronological vs biological age (i.e., accelerated vs decelerated aging). While first-generation clocks were based on chronological age (e.g., Hannum, Horvath 2013, Horvath 2018, Lin), second-generation clocks have been developed based on phenotypic age (e.g., PhenoAge), and lifespan (e.g., GrimAge) and third-generation clocks are based on the pace of aging (e.g., DunedinPoAm, DunedinPACE).

Aging clocks are not without controversy: there is no gold standard for measuring epigenetic biological aging. Aging-related biological changes may be correlates rather than causes of aging, the relationship between clocks and disease pathology is still in its infancy, and there is a current proliferation of clocks (Bell et al., 2019). Nonetheless, over the past decade, aging clocks have become widely adopted as important tools for understanding biological aging and have been redefining notions of “pro-longevity” lifestyles (Galkin et al., 2023). So, exploring aging clocks alongside other biological approaches could provide important complementary insight into the molecular underpinnings of health. As part of this, there is increasing interest in finding modifiable lifestyle factors that might have “anti-aging” effects. Avoiding smoking and binge drinking, maintaining a healthy body weight, adopting a Mediterranean diet, reducing stress, and engaging in meditation have all been demonstrated through combinations of experimental and epidemiological studies to reduce epigenetic age (Galkin et al., 2023).

However, this work is still at an early stage. Some leisure activities have never been studied at all. Arts and cultural engagement (ACEng) is increasingly recognized as a health behavior in its own right, comprising diverse “active ingredients” that are beneficial to health (e.g., social interaction, cognitive stimulation, multi-sensory stimulation, creativity, etc.) and activating complex psychological, biological, social and behavioral mechanisms of action that relate to mental and physical health outcomes (Fancourt & Finn, 2019; Fancourt et al., 2021). Experimental studies have already demonstrated that ACEng can affect gene regulation. For example, compared to relaxing, listening to music upregulates genes involved in processes such as dopamine secretion, enhanced synaptic function, and neurogenesis, alongside upregulating specific RNA proteins (microRNAs) that repress inflammatory cytokines and support neuronal and synaptic plasticity (Kanduri et al., 2015a, 2015b; Nair et al., 2019, 2021). Music has also been demonstrated to be more effective than other activities, such as reading newspapers, for reversing stress signatures in gene expression following laboratory-induced stressors (Bittman et al., 2005). However, there are no epidemiological studies of ACEng and epigenetic aging to date.

Even more prominent health-promoting leisure activities, such as exercise, have only received scant attention. Experimentally, physical activity (PA) has been demonstrated to cause DNAm changes. For example, people with a lifelong history of PA display lower DNAm levels on gene promoters in muscle tissue (Sailani et al., 2019), and interventions that increase PA reduce epigenetic mutation load (i.e., the total number of stochastic epigenetic mutations or outlier methylation patterns at CpG sites), which has been proposed as a complementary DNAm-based biomarker of healthy aging (Fiorito et al., 2021). Observationally, few studies have been conducted looking at PA and combined CpG sites within epigenetic clocks. Some very small studies (*n *< 250) have reported null findings when relating PA to individual epigenetic clocks (Fiorito et al., 2021; Sillanpää et al., 2019). Other studies have reported associations between PA and aspects of physical performance (e.g., grip strength and jumping height) and several epigenetic clocks, including PhenoAge, FitAge, and GrimAge (Jokai et al., 2023; Noroozi et al., 2024). However, these studies have failed to consider important confounders such as socioeconomic position (SEP), smoking, BMI, or blood cell compositions. Recently, analyses of larger cohort studies have shown more promising results. An analysis of adults in the Rhineland Study (*n *= 3,567) found that accelerometer-derived step count and both volume and intensity of PA were related to lower GrimAge and PhenoAge acceleration, but not Hannum or Horvath 2013 (Fox et al., 2023). And the U.S. Sister Study (*n *= 2,758) found that after adjusting for covariates, hours per week of leisure-time PA was only related to GrimAge but had no associations with Hannum, Horvath 2013, or PhenoAge (Kresovich et al., 2021).

However, some key challenges remain with the existing literature. First, studies have only focused on a limited number of aging clocks. Each aging clock defines biological age in its own way using a distinct set of CpG sites, different primary domains (tissue, health conditions, age range), different algorithms (target definition, machine learning model), and different target variables (chronological age, phenotypic age, time-to-death, pace of aging, etc.). Consequently, the associations between leisure activity and biological age understandably vary between clocks, meaning outcome-wide approaches using multiple aging clocks are important for drawing broader conclusions (Bell et al., 2019). Second, *how* leisure is conceptualized and defined is underexplored. While frequency is a standard metric of people’s engagement, a variety of engagement metrics may also be important. Variety provides people with greater opportunity to access different “active ingredients” of leisure - i.e., different patterns of cognitive, physical, and social stimuli, which may have different mechanistic pathways to biological aging (Warran et al., 2022). Additionally, social identity theory posits that engagement with multiple groups provides more diverse social identities, which can be crucial to psychological processes of stress-buffering, coping, and resilience (Hogg, 2016). Even when leisure is not overtly social, it can bring personal identities as being part of a collective that does that activity (e.g., “runner” or “artist”). Third, it is crucial to take into account diverse confounding factors. Previous analyses have largely relied on conditioning on confounders via simple regression adjustment. However, this leaves the potential for residual confounding imbalance. More sophisticated statistical approaches, such as doubly robust estimation, offer new opportunities for improving causal inference. Therefore, drawing data from a large nationally representative cohort study, this study aimed to assess the association of different measures ACEng and PA with seven different epigenetic clocks using a doubly robust statistical approach, providing new insight into the relationship between leisure and epigenetic aging.

## Materials and methods

### Data

Understanding Society, the UK Household Longitudinal Study (UKHLS), is a nationally representative panel survey of members of 40,000 private households in the UK. It was launched in 2009, with participants being followed up on annually. Detailed information on the sampling strategy can be found in the sampling design report (Lynn, 2009). We used data from the DNAm subsample. Between 2010 and 2012 (waves 2 and 3), blood samples were collected from adult participants during nurse visits. DNAm profiling was conducted from blood samples of 3,654 eligible individuals of white European ancestry who had consented to blood sampling and genetic analysis (wave 2: *n *= 1,425, wave 3: *n *= 2,229). Over 850,000 methylation sites across the genome were measured using the Illumina Methylation EPIC BeadChip. Data were pre-processed via quality control procedures, including outlier removal, filtering poor-quality probes, and quantile normalization (Bao et al., 2022). Exposures were obtained from the wave 2 adult survey (2010–2012), which included a special module on leisure activities. After excluding participants with missing data and outliers in outcome measures (3 standard deviations (SD) from the mean), we had an analytical sample of 3,556 (Supplementary Figure 1).

### Measures

#### Outcomes

UKHLS contains seven epigenetic clocks constructed from the DNAm data across three generations. First-generation clocks are trained exclusively on chronological age and include the single-tissue Hannum clock, Horvath 2013 (estimated from multiple tissues/cells) (Horvath, 2013), Horvath skin & blood (Horvath 2018) clock (another multi-tissue clock with improved accuracy on cultured cells) (Horvath et al., 2018), and Lin clock (based on DNAm profiles of 25 cancer types) (Lin & Wagner, 2015).

The second-generation clocks are trained on a composite measure of mortality and disease morbidity alongside chronological age. The one available in UKHLS is the PhenoAge clock, which is based on clinical biomarkers of phenotypic age (Levine et al., 2018).

The third-generation clocks are designed to quantify paces of biological aging rather than static status. The DunedinPoAm clock is considered the first of the third-generation clocks (Crimmins et al., 2024). It is trained on a composite measure of longitudinal changes over time in 18 biomarkers of blood chemistry and organ systems (Belsky et al., 2020). The DunedinPACE clock is an updated version of DunedinPoAm with longer follow-ups and more reliable DNA methylation probes (Belsky et al., 2022). While the first- and second-generation clocks above were measured in years, DunedinPoAm and DunedinPACE were measured in rates of biological age per chronological year.

#### Exposures

ACEng was measured by asking if participants had done anything in four sets of activities in the last 12 months (yes or no): (a) participatory arts (e.g., singing, dancing, painting, photographing, crafting), (b) receptive arts (e.g., attending art exhibitions/events), (c) visiting heritage sites (e.g., historic parks, historic buildings, monuments), (d) other cultural activities (e.g., going to museums, libraries or archives). Frequency of engagement was derived by using the highest frequency across the four sets of activities, originally recorded in five categories: at least once a week, at least once a month, at least three or four times a year, twice in the last 12 months, once in the last 12 months. Due to low response frequencies in the final two categories, we combined them into one category and recoded the variable into four categories, from lowest to highest frequency: once or twice yearly or less, three or four times yearly, monthly, weekly. We also derived an ACEng diversity measure by counting the number of activities and splitting this into quartiles: low (0–2), medium (3–6), high (7–10), and very high (≥11).

PA was measured by a list of sporting activities, including vigorous (e.g., running, swimming, boxing, cycling, football), moderate (e.g., skiing, racquet sports, angling/fishing, yoga/Pilates if age > 64), and mild (e.g., rambling, snooker, yoga/Pilates if age < 65) activities. PA frequency was derived by using the highest frequency between vigorous/moderate and mild activities, coded as no, <monthly, monthly, weekly. A PA diversity measure was derived by counting the number of activities, recoded into four categories: none, low (1), medium (2/3), and high (≥4). Furthermore, we also considered a self-rated PA activeness measure on a scale of 0 to 10, which was recoded into five categories based on the distribution of the original variable: not active (0), low (1–2), medium (3–4), high (5–6), and very high (7–10).

#### Covariates

We considered a range of demographic and socioeconomic covariates in the main analyses, including age (range 16–90), age-squared, sex (female, male), marital status (single, married/cohabitating, separated/divorced/widowed), living with children (yes, no), living area (rural, urban), education (no qualification, GCSE or below, A level or above, degree or above), household income quintiles, employment status (employed, other), and area deprivation quintiles. It is also essential to control for the gap between data collection dates between exposures and outcomes, given that the blood samples of 2,229 participants were collected in wave 3. In addition, we also adjusted for a set of technical covariates of various cell composition estimates (CD8-T cells, CD4-T cells, Natural Killer cells, B cells, monocytes, and granulocytes) (Bao et al., 2022). In sensitivity analyses, we accounted for additional behavioral and health factors that could act as both confounders and potential mediators on the causal pathway, including smoking (never smoker, ex-smoker, current smoker), drinking frequency (on a scale of 1—almost every day to 8—not at all in the last 12 months), self-reported long-standing physical or mental impairment, illness or disability (yes, no), and Body Mass Index (BMI, coded as: <25, ≥25 & <30, ≥30). All covariates were measured at wave 2, except for BMI, which was measured during nurse visits across waves 2 and 3.

### Statistical analysis

Data were analyzed using doubly robust estimation using the inverse-probability-weighted regression adjustment (IPWRA) estimator. This method involves building two models to account for non-random treatment assignment: (a) a regression adjustment model for the outcome and (b) a treatment-assignment model for the exposure. It uses weighted regression coefficients to compute averages of treatment-level predicted outcomes, where the weights are the estimated inverse probabilities obtained from the treatment-assignment model. IPWRA has the double-robust property: it only requires the outcome model or the treatment-assignment model to be correctly specified, not both (Wooldridge, 2010). Both models controlled for demographic and socioeconomic covariates described above. The outcome model additionally controlled for data collection gaps and technical covariates. The IPWRA model was fitted separately for each exposure (ACEng frequency, ACEng diversity, PA frequency, PA diversity). We conducted sensitivity analyses that (a) considered self-reported levels of activeness in PA, (b) additionally controlled for behavioral and health factors, and (c) restricted the sample to those aged 40 or above because it is suggested that aging is a non-linear process with the first substantial acceleration in the 40s (Shen et al., 2024). The outcome models were fitted using linear regression, and treatment-assignment models using multinomial logistic regression. All analyses were implemented in Stata 18.

## Results

### Descriptives

The average age of our analytical sample was 52.1 years compared to 47.5 years in the original sample, and there was an underrepresentation of single persons (10.9% vs 22.5%). However, the analytical sample was reasonably evenly distributed across household income quintiles, and the distributions of other demographic, socioeconomic, behavioral, and health factors were largely similar to the original sample (Table 1).

**Table 1:** Analytical sample characteristics compared to the original wave 2 sample.

Variables	Analytical sample (*n *= 3,556)	Original sample (*n *= 46,891)
	%/mean (SD)	**%/mean (SD)** ^a^
**Sex**		
Male	44.0	48.3
Female	56.0	51.7
Age	52.1 (15.3)	47.5 (18.9)
**Living with children**		
Yes	28.4	30.3
No	71.6	69.7
**Marital status**		
Single	10.9	22.5
Married/cohabitating	72.9	63.7
Separated/divorced/widowed	16.2	13.8
**Household income**		
Q1—lowest	17.9	19.4
Q2	19.2	19.3
Q3	20.7	19.3
Q4	21.6	20.1
Q5—highest	20.7	21.9
**Area of living**		
Urban	71.8	75.9
Rural	28.2	24.1
**Educational qualification**		
No qualification	12.8	14.6
GCSE or below	33.5	31.7
A level or above	32.3	32.4
Degree or above	21.5	21.4
**Employment status**		
Employed	58.5	57.6
Other	41.5	42.4
**Area deprivation**		
Q1—most deprived	14.4	17.6
Q2	18.2	18.8
Q3	22.7	21.4
Q4	22.1	21.1
Q5—least deprived	22.5	21.0

*Note*. GCSE = General Certificate of Secondary Education.

a Wave 2 cross-sectional weights were applied for the original sample.

ACEng was relatively common among participants, with 82% of people doing three or more activities and 27.9% engaging in 11 or more activities (Table 2). More than three-quarters of people engaged in ACEng monthly or weekly.

**Table 2:** Descriptive statistics of the exposure and outcome variables (*n *= 3,556).

Variables	%/mean (SD)	Min-max
**ACEng frequency**		
≤1/2 yearly	10.5	–
3+ yearly	12.3	–
Monthly	16.8	–
Weekly	60.3	–
**ACEng diversity**		
Low: 0–2	18.3	–
Medium: 3–6	30.0	–
High:7–10	23.7	–
Very high: ≥11	27.9	–
**PA frequency**		
None	18.8	–
< Monthly	16.0	–
Monthly	15.6	–
Weekly	49.7	–
**PA diversity**		
None	19.3	–
Low: 1	20.3	–
Medium: 2–3	28.6	–
High: ≥4	31.8	–
**PA activeness**		
Not active	23.5	–
Low	18.5	–
Medium	19.8	–
High	19.8	–
Very high	18.4	–
Hannum	50.7 (11.1)	19.9–81.2
Horvath 2013	57.3 (10.5)	26.6–87.5
Horvath 2018	50.4 (12.5)	15.0–87.5
Lin	46.1 (12.3)	10.3–84.0
PhenoAge	45.1 (12.4)	7.8–82.5
DunedinPoAm	1.0 (0.1)	0.8–1.2
DunedinPACE	1.1 (0.1)	0.7–1.5

*Note.* ACEng = arts and cultural engagement

PA had a relatively low diversity: 19.3% of participants did not do any PA, and less than a third engaged in four or more activities. However, PA frequency was higher, with nearly half of the participants engaging weekly. PA activeness was roughly evenly distributed in quintiles.

### ACEng and epigenetic aging

**Figure 1:**
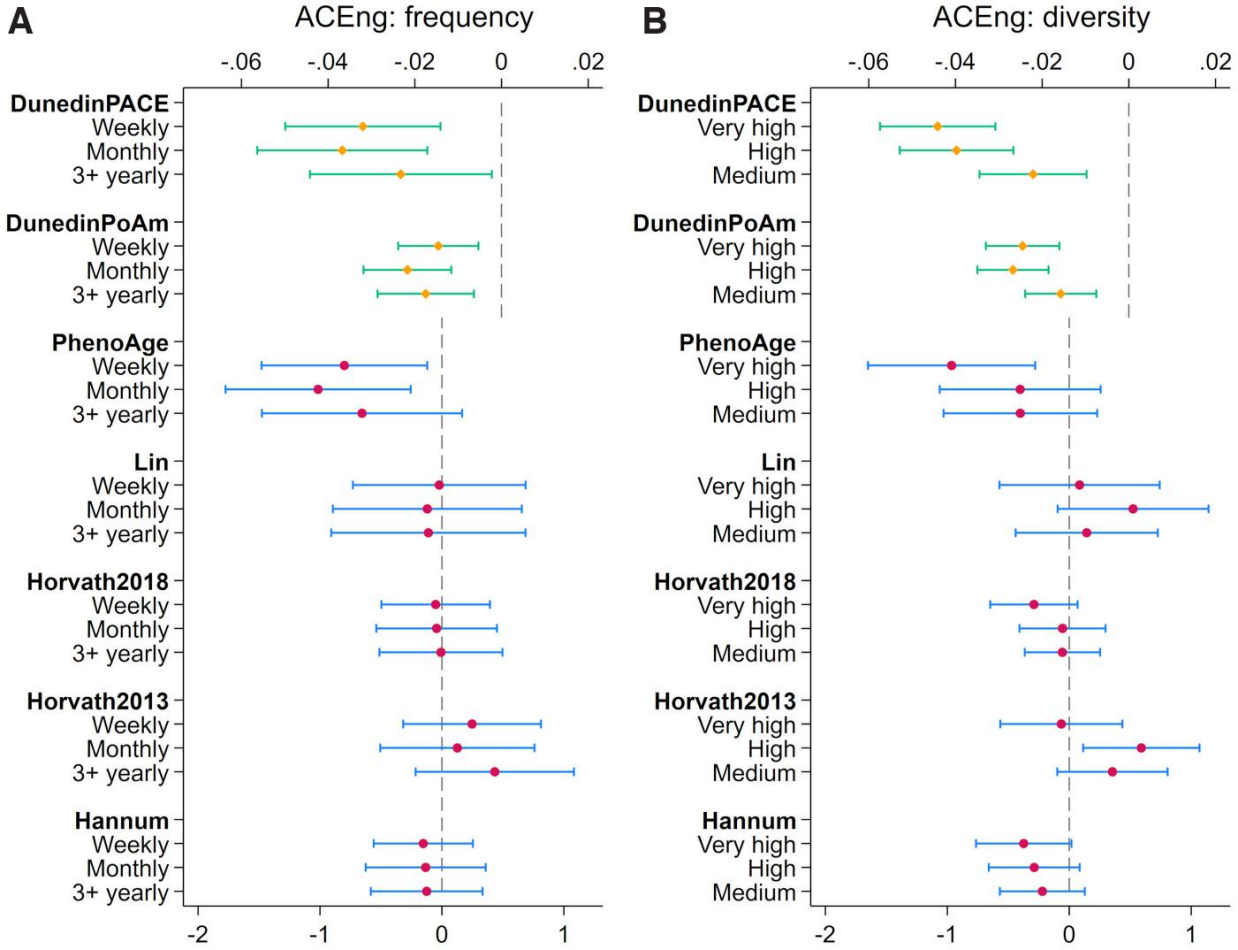
Estimated average treatment effect and 95% confidence intervals for ACEng frequency (A) and diversity (B) measures from doubly robust estimation using the inverse-probability-weighted regression adjustment (IPWRA) estimator (*n *= 3,556). ACEng = arts and cultural engagement.

For frequency of ACEng, there was no evidence of association with any of the first-generation clocks (Figure 1A and Supplementary Table 1). But associations were found with the second-generation PhenoAge clock and the third-generation DunedinPoAm and DunedinPACE clocks. For PhenoAge, although no evidence was found for the difference between the two low-frequency groups, epigenetic aging was 0.8 years lower in people engaging weekly (95% CI=[−1.48, −0.12], *p *= .021), and 1.02 years lower in people engaging monthly (95% CI=[−1.77, −0.26], *p *< .001) compared to one or two times yearly. For DunedinPoAm, ACEng frequency of at least three times yearly (95% CI=[−0.03, −0.01], *p *= .002), monthly (95% CI=[−0.03, −0.01], *p *< .001) and weekly (95% CI=[−0.02, −0.01], *p *= .002) were associated with a slower pace of epigenetic aging by 0.01 to 0.02 biological years per one chronological year. The DunedinPACE coefficients were larger in magnitude than those for DunedinPoAm. Engagement at least three times yearly was associated with a slower pace of epigenetic aging by 0.02 (95% CI=[−0.04, −0.002], *p *= .03), engagement monthly by 0.04 (95% CI=[−0.06, −0.02], *p *< .001), and weekly by 0.03 (95% CI=[−0.05,−0.01], *p *< .001).

For diversity of ACEng, there was again little evidence of association with the first-generation clocks. However, there was some evidence that the highest level of diversity was associated with a lower value of the second-generation PhenoAge clock (ATE = −0.96 years lower, 95% CI = [−1.65, −0.28], *p *= .006) (Figure 1B and Supplementary Table 1). For the third-generation DunedinPoAm clock, higher diversity levels were associated with a slower pace of epigenetic aging by 0.02 to 0.03. Similarly, for DunedinPACE, higher diversity levels were associated with a slower pace by 0.02 to 0.04.

### PA and epigenetic aging

**Figure 2:**
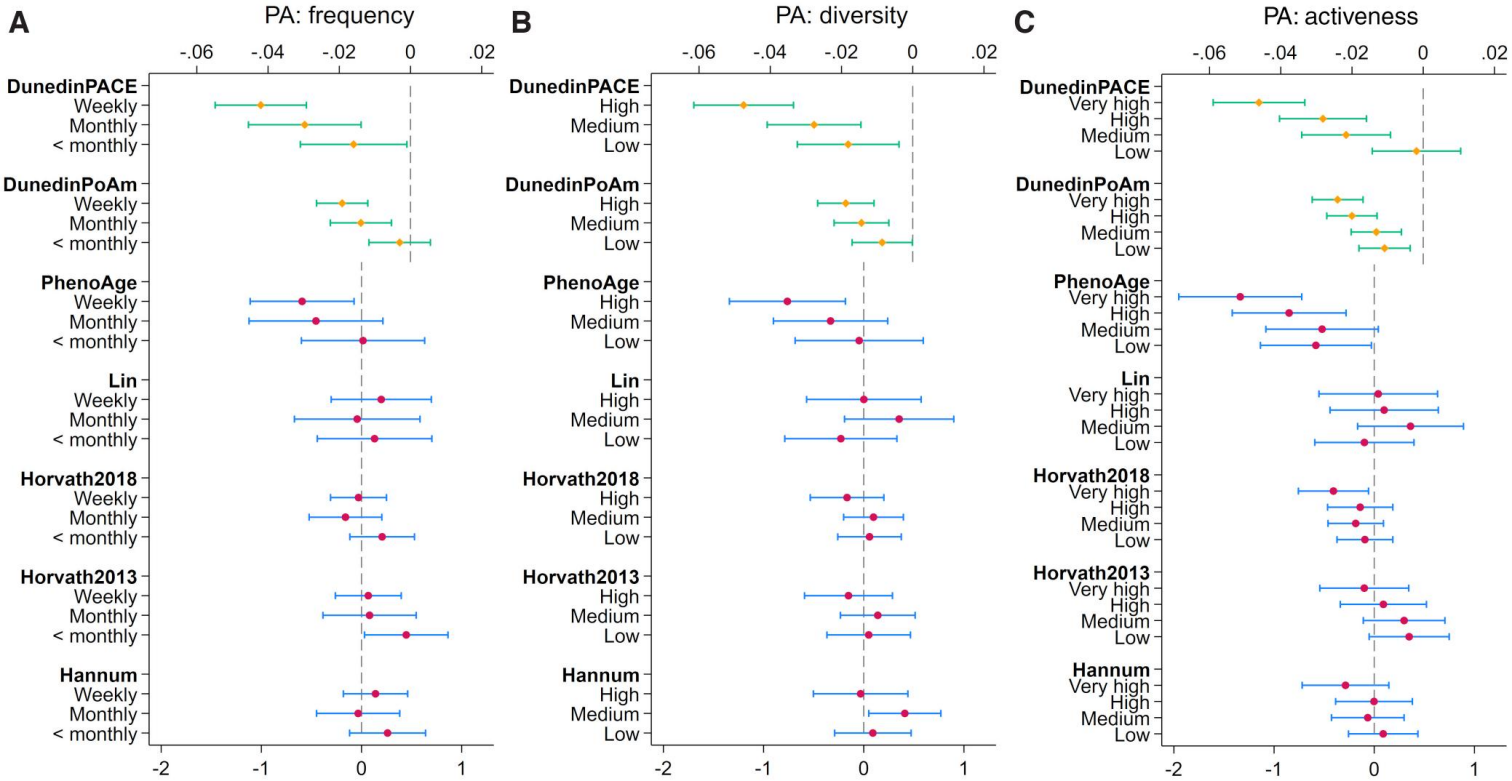
Estimated average treatment effect and 95% confidence intervals for PA frequency (A), diversity (B), and activeness (C) from doubly robust estimation using the inverse-probability-weighted regression adjustment (IPWRA) estimator (*n *= 3,556). PA = physical activity.

For frequency of PA, there was no evidence of association with any of the first-generation clocks (Figure 2A and Supplementary Table 2). However, for PhenoAge, epigenetic aging was 0.59 years lower in people engaging weekly (95% CI=[−1.11, −0.07], *p *= .025). For the DunedinPoAm clock, although no evidence was found for less than monthly engagement, monthly engagement was associated with a slower pace of epigenetic aging by 0.01 (95% CI=[−0.02, −0.01], *p *= .001), and weekly engagement by 0.02 (95% CI=[−0.03, −0.01], *p *< .001). For DunedinPACE, evidence was found for all higher frequency levels compared to no engagement, with a slower pace by 0.02 to 0.04.

For diversity of PA, there was again little evidence of association with the first-generation clocks (Figure 2B and Supplementary Table 2). Some evidence was found for the PhenoAge clock: the highest level of diversity was associated with a lower epigenetic aging by 0.76 years (95% CI=[−1.34, −0.18], *p *= .01). For DunedinPoAm, all low, medium, and high diversity levels were associated with a slower pace of epigenetic aging by 0.01 to 0.02 compared to no PA. Similarly, for DunedinPACE, all diversity levels were associated with a slower pace of epigenetic aging, by 0.02 to 0.05.

### Sensitivity analyses

There was little evidence of associations in first-generation clocks for levels of activeness (Figure 2C and Supplementary Table 2). However, high levels of PA activeness were associated with 0.85 years lower PhenoAge (95% CI=[−1.42, −0.28], *p* = .003), and very high levels of PA activeness with 1.34 years lower PhenoAge (95% CI=[−1.95, −0.72], *p *< .001). And for DunedinPoAm, all activeness levels, from low to very high, were associated with a slower pace of epigenetic aging, by 0.01 to 0.02. For DunedinPACE, no difference was found between low and no engagement, but medium (ATE=−0.02, 95% CI=[−0.03, −0.01], *p *= .001), high (ATE=−0.03, 95% CI=[−0.04, −0.02], *p *< .001) and very high (ATE=−0.05, 95% CI=[−0.06, −0.03], *p *< .001) levels of activeness were all associated with a slower pace of epigenetic aging.

The associations of ACEng and PA with PhenoAge, DunedinPoAm, and DunedinPACE largely persisted even after accounting for behavioral and health factors (Supplementary Tables 3 and 4). The results from sensitivity analyses restricting the sample to those aged 40 or above were consistent with the main results, with generally larger effect sizes (Supplementary Tables 5 and 6).

## Discussion

Using an outcome-wide approach involving seven epigenetic clocks, we found associations between two health-promoting leisure activities—ACEng and PA—and slower epigenetic aging. Specifically, ACEng and PA were related to PhenoAge, DunedinPoAm and DunedinPACE clocks, although not to the other measured clocks (Lin, Horvath 2018, Horvath 2013, and Hannum), with comparable effect sizes between ACEng and PA. Evidence was consistently found across different measures of engagement, including diversity and frequency for ACEng, as well as frequency, diversity, and activeness for PA. These findings were generally stronger amongst middle-aged and older adults aged 40 or above.

This was the first study to show a relationship between ACEng and epigenetic aging. It builds on strong theoretical and empirical underpinnings for why the arts could affect fundamental biological hallmarks of aging. Life-course psychosocial stressors have been clearly linked with accelerated epigenetic aging and broader physiological wear and tear across tissues and organ systems (Zannas, 2016). One of the fundamental mechanistic effects of arts engagement is reductions in psychophysiological markers of stress, demonstrated in clinical and non-clinical studies (de Witte et al., 2022; Finn & Fancourt, 2018; Lee et al., 2023). Notably, a variety of engagement has been proposed as key here as it provides opportunities for diverse exposure to active ingredients, formation of multiple identities, and even increased social capital (i.e., tangible and intangible resources), all of which support buffering of stressors (Cohen & Wills, 1985). So, it is significant that variety and frequency were both related to slower epigenetic aging. For both ACEng and PA, reductions in inflammatory pathways (which are well-reported for both) (Fancourt et al., 2016a, 2016b; Silverman & Deuster, 2014) may also be important mechanisms between engagement and epigenetic alterations. Anti-inflammatory effects of ACEng and PA engagement have been linked to methylation status as well as being a hallmark of aging (“inflammaging”) (Zhu et al., 2021). Additionally, improvements in cardiovascular risk have been demonstrated to be mediators of the link between PA and epigenetic aging (Fox et al., 2023), and this may also be the case for ACEng, for which there is strong mechanistic evidence of benefits for diverse cardiometabolic traits (Cao & Zhang, 2023; McCrary & Altenmüller, 2021; Peng et al., 2020). Notably, the findings were independent of behavioral and health factors, including smoking and BMI, which is important given that they have been strongly linked to epigenetic age both observationally and experimentally (Galkin et al., 2023).

Notably, we only found results for so-called “second-generation” and “third-generation” clocks, but not for “first-generation” clocks. This echoes some previous studies. The Rhineland study found results for second-generation clocks (PhenoAge and GrimAge) but not for first-generation clocks (Horvath 2013 and Hannum), and a study using The Irish Longitudinal Study of Ageing (TILDA) found associations between physical performance (walking speed) and second- but not first-generation clocks (Fox et al., 2023; McCrory et al., 2021). Null findings for first-generation clocks have previously been shown for measures related to physical performance in multiple previous studies (Maddock et al., 2020; Quach et al., 2017). There are several reasons why second- and third-generation clocks may be more relevant to picking up decelerated aging associated with leisure engagement. First-generation clocks are generally less sensitive predictors of age-related decline in clinical health measures (Horvath & Raj, 2018). This is because they do not incorporate clinical biomarkers in their derivation and hence are less sensitive to capturing the epigenetic aging deceleration that results from biobehavioral factors such as protective health behaviors (McCrory et al., 2020). First-generation clocks were also trained on cross-sectional data, which, unlike longitudinal data, do not account for mortality selection. This biases the algorithm to select markers that are correlative with aging rather than causal, because causal loci that should exhibit diminishing age prediction in later life as the individuals exhibiting these traits are progressively selected out of the cohort (Nelson et al., 2020). By using multiple different generations of aging clocks, our study provides a clearer demonstration of the differential findings between earlier and more recent clocks.

Our study has many strengths, including using a representative cohort study, rich measures of both frequency and diversity of behaviors for our two leisure activities, adoption of an outcome-wide approach to epigenetic clocks, and consideration of diverse confounding factors. However, there are some limitations. First, we relied on participants’ self-reports on their behaviors, which brings the risk of recall bias and self-report bias. However, some of our clocks overlapped with the Rhineland study, and were corroborated, which is reassuring given that their study used objective assessments of behaviors. Second, although we included all identified confounding factors, including a particular focus on diverse measures of SEP, unidentified or unmeasured confounding remains a risk. Nonetheless, we adopted a doubly robust estimation approach (a methodological advance on previous work relating lifestyle factors to epigenetic clocks), which allows for misspecification of confounders for either the exposure or the outcome. Third, we relied on DNAm present in whole blood. However, the effect of leisure on the epigenome is likely not uniform across the body. This is particularly important for PA, where aging deceleration may be different in, say, muscle tissues. So, future studies are needed to expand the investigation to include a wider range of epigenetic clocks beyond the clocks examined in the present study. In particular, studies focusing on more specific DNAm tissue data are encouraged. Finally, we acknowledge that the data used in this study were collected over a decade ago, and patterns of ACEng and PA in the population may have shifted to some extent. However, the fundamental relationships of ACEng and PA with epigenetic aging, as well as their confounding structure and underlying biological mechanisms, are unlikely to have changed in nature. Our findings, therefore, still hold scientific and public health relevance.

Overall, our results provide the first evidence that ACEng, a much more recently recognized health behavior, is related to epigenetic aging, suggesting the value of its exploration alongside other lifestyle factors (Galkin et al., 2023). In particular, diversity of engagement appears as important as frequency of engagement. It is also of note that the effect size was comparable for ACEng and for PA with respect to epigenetic aging. Our findings add to the existing literature on arts and health, positioning ACEng as a potential contributor to healthy aging at the biological level and underscoring the potential value of integrating ACEng into public health strategies and initiatives. We also extend the existing understanding of the relationship between PA and epigenetic aging, demonstrating a clear association with second- and third- but not first-generation epigenetic clocks and extending analyses to new clocks not included in previous analyses. Our findings are relevant for several reasons. First, decelerations in aging clocks, including those within our study, have been demonstrated to have clinical (as well as statistical) importance, including improvements in physical performance, polypharmacy, cognitive state, and all-cause mortality risk (McCrory et al., 2021). Indeed, our strongest results involved DunedinPACE, which has shown improved performance than DunedinPoAm and has been related to improved performance in physical and physiological measures of aging over subsequent years (Belsky et al., 2022). And it is notable that associations between leisure and epigenetic aging became more prominent in adults from mid-life. Second, recent work suggests that epigenetic aging is potentially reversible (Fahy et al., 2019). The persistence of epigenetic changes in response to modifiable behaviors, such as leisure engagement, is greatly underexplored. However, given the experimental evidence reviewed earlier on the effects of both ACEng and PA on DNAm generally and epigenetic clocks specifically (Fiorito et al., 2021; Kanduri et al., 2015b; Nair et al., 2021), future intervention studies could explore whether lifestyle changes have any value to slowing or potentially reversing epigenetic aging.

## Supplementary Material

igag038_Supplementary_Data

## Data Availability

This study was not preregistered. Data of this study are publicly available via the UK Data Service: https://ukdataservice.ac.uk/
