# Supplementary material for: Does leisure activity matter for epigenetic aging? Analyses of arts engagement and physical activity in the UK Household Longitudinal Study
Source: Innov Aging. 2026 May 11;10(6):igag038. doi: 10.1093/geroni/igag038 (PMC13189860; doi:10.1093/geroni/igag038)
Supplement: igag038_Supplementary_Data [file igag038_supplementary_data.pdf]

Innovation in Aging **Supplementary Material: Fancourt, Masebo, Finn, Mak, & Bu. Does leisure activity matter for epigenetic ageing? Analyses of arts engagement and physical activity in the UK Household Longitudinal Study.**

Figure S1. Sample selection diagram

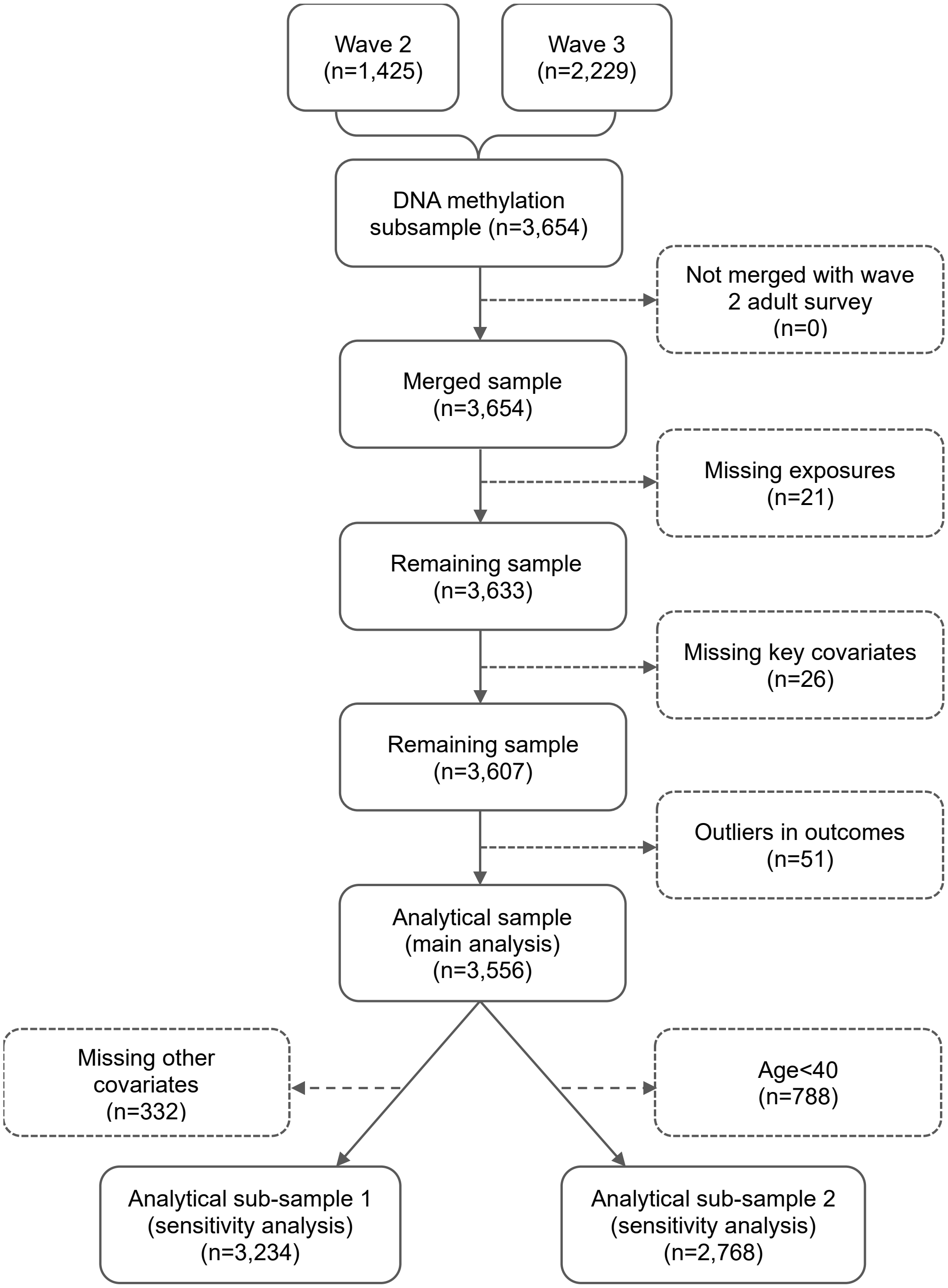

Table S1 Estimated average treatment effect, 95% confidence interval and p value for ACEng frequency and diversity measures from doubly robust estimation using IPWRA (main analysis, n=3,556)

	**Hannum**	**Horvath2013**	**Horvath2018**	**Lin**	**PhenoAge**	**DunedinPoAm**	**DunedinPACE**
Frequency:							
3+ yearly	-0.12	0.43	-0.01	-0.11	-0.66	-0.02	-0.02
	[-0.58, 0.33]	[-0.22, 1.08]	[-0.50, 0.50]	[-0.91, 0.69]	[-1.48, 0.17]	[-0.03, -0.01]	[-0.04, -0.002]
	0.593	0.191	0.975	0.785	0.118	0.002	0.030
Monthly	-0.13	0.13	-0.04	-0.12	-1.02	-0.02	-0.04
	[-0.63, 0.36]	[-0.51, 0.76]	[-0.54, 0.45]	[-0.89, 0.66]	[-1.77, -0.26]	[-0.03, -0.01]	[-0.06, -0.02]
	0.596	0.694	0.863	0.762	0.009	<0.001	<0.001
Weekly	-0.15	0.25	-0.05	-0.02	-0.80	-0.01	-0.03
	[-0.56, 0.25]	[-0.32, 0.81]	[-0.50, 0.39]	[-0.73, 0.69]	[-1.48, -0.12]	[-0.02, -0.01]	[-0.05, -0.01]
	0.462	0.391	0.821	0.952	0.021	0.002	<0.001
Diversity:							
Medium	-0.22	0.35	-0.06	0.14	-0.40	-0.02	-0.02
	[-0.57, 0.13]	[-0.10, 0.81]	[-0.36, 0.25]	[-0.44, 0.73]	[-1.03, 0.23]	[-0.02, -0.01]	[-0.03, -0.01]
	0.215	0.125	0.722	0.630	0.212	<0.001	<0.001
High	-0.29	0.59	-0.05	0.52	-0.40	-0.03	-0.04
	[-0.66, 0.09]	[0.11, 1.07]	[-0.41, 0.30]	[-0.10, 1.14]	[-1.06, 0.26]	[-0.03, -0.02]	[-0.05, -0.03]
	0.131	0.015	0.761	0.097	0.232	<0.001	<0.001
Very high	-0.37	-0.06	-0.29	0.08	-0.96	-0.02	-0.04
	[-0.76, 0.02]	[-0.57, 0.44]	[-0.65, 0.07]	[-0.57, 0.74]	[-1.65, -0.28]	[-0.03, -0.02]	[-0.06, -0.03]
	0.061	0.800	0.113	0.801	0.006	<0.001	<0.001

Notes: The treatment-assignment models controlled for socio-demographic factors (age, age-squared, sex, marital status, living with children, living area, education, household income, employment status, and area deprivation). The outcome model additionally controlled for gap and technical covariates of cell composition.

Table S2 Estimated average treatment effect, 95% confidence interval and p value for PA frequency, diversity and activeness measures from doubly robust estimation using IPWRA (main analysis, n=3,556)

	**Hannum**	**Horvath2013**	**Horvath2018**	**Lin**	**PhenoAge**	**DunedinPoAm**	**DunedinPACE**
Frequency:							
< monthly	0.26	0.45	0.21	0.13	0.02	-0.00	-0.02
	[-0.12, 0.64]	[0.03, 0.86]	[-0.12, 0.53]	[-0.44, 0.70]	[-0.60, 0.63]	[-0.01, 0.01]	[-0.03, -0.001]
	0.179	0.036	0.211	0.655	0.962	0.490	0.036
Monthly	-0.03	0.08	-0.16	-0.04	-0.45	-0.01	-0.03
	[-0.45, 0.38]	[-0.39, 0.55]	[-0.52, 0.20]	[-0.67, 0.58]	[-1.12, 0.21]	[-0.02, -0.01]	[-0.05, -0.01]
	0.874	0.735	0.387	0.892	0.182	0.001	<0.001
Weekly	0.14	0.07	-0.03	0.20	-0.59	-0.02	-0.04
	[-0.18, 0.46]	[-0.26, 0.40]	[-0.31, 0.25]	[-0.30, 0.70]	[-1.11, -0.07]	[-0.03, -0.01]	[-0.05, -0.03]
	0.393	0.688	0.834	0.440	0.025	<0.001	<0.001
Diversity:							
Low	0.09	0.05	0.06	-0.23	-0.05	-0.01	-0.02
	[-0.29, 0.47]	[-0.37, 0.47]	[-0.26, 0.37]	[-0.79, 0.33]	[-0.69, 0.59]	[-0.02, -0.001]	[-0.03, -0.004]
	0.641	0.816	0.722	0.424	0.888	0.047	0.013
Medium	0.41	0.14	0.10	0.35	-0.33	-0.01	-0.03
	[0.05, 0.77]	[-0.23, 0.51]	[-0.20, 0.40]	[-0.19, 0.90]	[-0.90, 0.24]	[-0.02, -0.01]	[-0.04, -0.01]
	0.026	0.462	0.522	0.204	0.255	<0.001	<0.001
High	-0.03	-0.15	-0.17	0.00	-0.76	-0.02	-0.05
	[-0.50, 0.44]	[-0.59, 0.29]	[-0.53, 0.20]	[-0.57, 0.57]	[-1.34, -0.18]	[-0.03, -0.01]	[-0.06, -0.03]
	0.897	0.496	0.374	0.996	0.010	<0.001	<0.001
Activity							
Low	0.09	0.35	-0.09	-0.10	-0.58	-0.01	-0.002
	[-0.26, 0.44]	[-0.05, 0.75]	[-0.37, 0.19]	[-0.59, 0.40]	[-1.14, -0.03]	[-0.02, -0.004]	[-0.01, 0.01]
	0.612	0.087	0.512	0.697	0.039	0.003	0.766
Medium	-0.06	0.30	-0.18	0.36	-0.52	-0.01	-0.02
	[-0.43, 0.30]	[-0.11, 0.71]	[-0.46, 0.09]	[-0.17, 0.89]	[-1.08, 0.04]	[-0.02, -0.01]	[-0.03, -0.01]

	Hannum	Horvath2013	Horvath2018	Lin	PhenoAge	DunedinPoAm	DunedinPACE
	0.726	0.151	0.191	0.179	0.069	<0.001	0.001
High	-0.00	0.09	-0.14	0.10	-0.85	-0.02	-0.03
	[-0.39, 0.38]	[-0.34, 0.52]	[-0.47, 0.19]	[-0.44, 0.64]	[-1.42, -0.28]	[-0.03, -0.01]	[-0.04, -0.02]
	0.991	0.677	0.399	0.720	0.003	<0.001	<0.001
Very high	-0.29	-0.10	-0.41	0.04	-1.34	-0.02	-0.05
	[-0.72, 0.15]	[-0.54, 0.34]	[-0.76, -0.06]	[-0.55, 0.63]	[-1.95, -0.72]	[-0.03, -0.02]	[-0.06, -0.03]
	0.194	0.660	0.022	0.895	<0.001	<0.001	<0.001

Notes: The treatment-assignment models controlled for socio-demographic factors (age, age-squared, sex, marital status, living with children, living area, education, household income, employment status, and area deprivation). The outcome model additionally controlled for gap and technical covariates of cell composition.

Table S3 Estimated average treatment effect, 95% confidence interval and p value for ACEng frequency and diversity measures from doubly robust estimation using IPWRA (sensitivity analysis with additional controls, n=3,234)

	**Hannum**	**Horvath2013**	**Horvath2018**	**Lin**	**PhenoAge**	**DunedinPoAm**	**DunedinPACE**
Frequency:							
3+ yearly	-0.21	0.50	-0.12	-0.03	-0.48	-0.01	-0.01
	[-0.68, 0.26]	[-0.12, 1.12]	[-0.63, 0.38]	[-0.84, 0.77]	[-1.25, 0.28]	[-0.02, -0.001]	[-0.03, 0.004]
	0.376	0.115	0.634	0.935	0.216	0.031	0.123
Monthly	-0.19	0.30]	-0.01	-0.08	-0.65	-0.01	-0.02
	[-0.66, 0.29]	[-0.30, 0.91]	[-0.49, 0.47]	[-0.86, 0.69]	[-1.35, 0.05]	[-0.02, -0.004]	[-0.04, -0.004]
	0.445	0.327	0.979	0.836	0.068	0.002	0.018
Weekly	-0.23	0.31	-0.11	-0.06	-0.65	-0.00	-0.02
	[-0.65, 0.19]	[-0.23, 0.86]	[-0.56, 0.33]	[-0.77, 0.65]	[-1.28, -0.03]	[-0.01, 0.00]	[-0.04, -0.004]
	0.279	0.259	0.613	0.863	0.040	0.134	0.016
Diversity:							
Medium	-0.10	0.39	0.07	-0.07	-0.30	-0.004	-0.01
	[-0.46, 0.27]	[-0.08, 0.86]	[-0.27, 0.41]	[-0.68, 0.54]	[-0.90, 0.30]	[-0.01, 0.003]	[-0.02, 0.004]
	0.596	0.105	0.702	0.818	0.326	0.281	0.190
High	-0.30	0.61	0.02	0.26	-0.14	-0.01	-0.02
	[-0.70, 0.09]	[0.10, 1.12]	[-0.37, 0.40]	[-0.39, 0.91]	[-0.77, 0.49]	[-0.02, -0.002]	[-0.03, -0.003]
	0.136	0.018	0.935	0.437	0.660	0.010	0.015
Very high	-0.21	-0.02	-0.08	-0.14	-0.68	-0.01	-0.03
	[-0.61, 0.18]	[-0.54, 0.50]	[-0.46, 0.30]	[-0.81, 0.52]	[-1.33, -0.03]	[-0.02, -0.001]	[-0.04, -0.01]
	0.285	0.949	0.676	0.678	0.041	0.033	<0.001

Notes: The treatment-assignment models controlled for socio-demographic (age, age-squared, sex, marital status, living with children, living area, education, household income, employment status, and area deprivation), behavioural and health factors (smoking, drinking, self-reported health status, BMI). The outcome model additionally controlled for gap and technical covariates of cell composition.

Table S4 Estimated average treatment effect, 95% confidence interval and p value for PA frequency, diversity and activeness measures from doubly robust estimation using IPWRA (sensitivity analysis with additional controls, n=3,234)

	**Hannum**	**Horvath2013**	**Horvath2018**	**Lin**	**PhenoAge**	**DunedinPoAm**	**DunedinPACE**
Frequency:							
< monthly	0.15	0.37	0.24	0.03	0.02	-0.001	-0.02
	[-0.25, 0.54]	[-0.10, 0.84]	[-0.11, 0.59]	[-0.57, 0.64]	[-0.64, 0.67]	[-0.01, 0.01]	[-0.03, -0.003]
	0.474	0.120	0.183	0.916	0.963	0.739	0.018
Monthly	-0.18	-0.13	-0.12	-0.35	-0.66	-0.01	-0.02
	[-0.62, 0.26]	[-0.63, 0.37]	[-0.52, 0.27]	[-1.00, 0.29]	[-1.35, 0.03]	[-0.01, 0.001]	[-0.04, -0.01]
	0.419	0.612	0.545	0.286	0.059	0.081	0.001
Weekly	0.23	0.12	0.12	0.11	-0.36	-0.01	-0.03
	[-0.11, 0.56]	[-0.25, 0.48]	[-0.18, 0.42]	[-0.41, 0.64]	[-0.91, 0.19]	[-0.02, -0.003]	[-0.04, -0.01]
	0.192	0.534	0.424	0.670	0.200	0.002	<0.001
Diversity:							
Low	0.07	-0.06	0.01	-0.43	0.04	-0.01	-0.02
	[-0.35, 0.49]	[-0.52, 0.39]	[-0.33, 0.35]	[-1.02, 0.16]	[-0.64, 0.72]	[-0.01, 0.001]	[-0.03, -0.004]
	0.748	0.793	0.957	0.155	0.909	0.110	0.010
Medium	0.38	0.11	0.16	0.17	-0.12	-0.01	-0.02
	[-0.00, 0.76]	[-0.30, 0.52]	[-0.16, 0.47]	[-0.40, 0.73]	[-0.70, 0.45]	[-0.01, -0.0001]	[-0.03, -0.01]
	0.053	0.593	0.323	0.569	0.673	0.045	0.001
High	-0.08	-0.33	-0.09	0.10	-0.61	-0.01	-0.03
	[-0.52, 0.37]	[-0.80, 0.15]	[-0.46, 0.28]	[-0.49, 0.69]	[-1.23, -0.00]	[-0.01, -0.001]	[-0.04, -0.01]
	0.738	0.182	0.624	0.745	0.049	0.034	<0.001
Activity							
Low	-0.06	0.31	-0.17	-0.25	-0.60	-0.005	0.001
	[-0.45, 0.33]	[-0.13, 0.75]	[-0.47, 0.13]	[-0.79, 0.28]	[-1.19, -0.01]	[-0.01, 0.001]	[-0.01, 0.01]
	0.750	0.165	0.272	0.357	0.048	0.099	0.931
Medium	-0.17	0.26	-0.17	0.30	-0.55	-0.01	-0.01
	[-0.57, 0.22]	[-0.17, 0.69]	[-0.46, 0.13]	[-0.25, 0.85]	[-1.13, 0.03]	[-0.01, -0.001]	[-0.02, -0.001]

	Hannum	Horvath2013	Horvath2018	Lin	PhenoAge	DunedinPoAm	DunedinPACE
	0.388	0.239	0.274	0.290	0.062	0.030	0.029
High	0.10	0.26	-0.01	0.26	-0.44	-0.01	-0.01
	[-0.32, 0.52]	[-0.20, 0.73]	[-0.35, 0.33]	[-0.31, 0.84]	[-1.03, 0.16]	[-0.02, -0.004]	[-0.02, 0.001]
	0.636	0.260	0.963	0.370	0.148	0.001	0.076
Very high	-0.12	-0.06	-0.17	-0.03	-0.92	-0.01	-0.02
	[-0.59, 0.35]	[-0.53, 0.41]	[-0.53, 0.20]	[-0.66, 0.59]	[-1.61, -0.23]	[-0.02, -0.01]	[-0.03, -0.01]
	0.607	0.797	0.368	0.921	0.009	<0.001	0.002

Notes: The treatment-assignment models controlled for socio-demographic (age, age-squared, sex, marital status, living with children, living area, education, household income, employment status, and area deprivation), behavioural and health factors (smoking, drinking, self-reported health status, BMI). The outcome model additionally controlled for gap and technical covariates of cell composition.

Table S5 Estimated average treatment effect, 95% confidence interval and p value for ACEng frequency and diversity measures from doubly robust estimation using IPWRA (sensitivity analysis: age≥40, n=2,768)

	**Hannum**	**Horvath2013**	**Horvath2018**	**Lin**	**PhenoAge**	**DunedinPoAm**	**DunedinPACE**
Frequency:							
3+ yearly	-0.03	0.39	0.23	0.19	-0.88	-0.02	-0.02
	[-0.56, 0.50]	[-0.23, 1.01]	[-0.29, 0.76]	[-0.62, 1.00]	[-1.78, 0.03]	[-0.04, -0.01]	[-0.05, -0.001]
	0.902	0.217	0.389	0.644	0.057	<0.001	0.038
Monthly	-0.16	-0.08	0.12	0.08	-1.20	-0.03	-0.04
	[-0.73, 0.41]	[-0.68, 0.53]	[-0.38, 0.63]	[-0.70, 0.87]	[-2.02, -0.38]	[-0.04, -0.02]	[-0.06, -0.02]
	0.592	0.800	0.632	0.835	0.004	<0.001	<0.001
Weekly	-0.16	0.08	0.15	0.31	-1.03	-0.02	-0.04
	[-0.62, 0.30]	[-0.41, 0.57]	[-0.27, 0.58]	[-0.38, 1.00]	[-1.74, -0.33]	[-0.03, -0.01]	[-0.05, -0.02]
	0.491	0.747	0.476	0.378	0.004	<0.001	<0.001
Diversity:							
Medium	-0.30	0.14	-0.27	0.06	-0.70	-0.02	-0.03
	[-0.70, 0.09]	[-0.31, 0.59]	[-0.62, 0.08]	[-0.61, 0.74]	[-1.39, -0.00]	[-0.03, -0.02]	[-0.04, -0.02]
	0.133	0.542	0.132	0.858	0.048	<0.001	<0.001
High	-0.41	0.27	-0.23	0.40	-0.52	-0.03	-0.05
	[-0.82, -0.00]	[-0.20, 0.74]	[-0.61, 0.14]	[-0.30, 1.10]	[-1.23, 0.20]	[-0.04, -0.02]	[-0.06, -0.03]
	0.048	0.266	0.223	0.258	0.160	<0.001	<0.001
Very high	-0.47	-0.29	-0.44	0.07	-1.17	-0.03	-0.05
	[-0.92, -0.01]	[-0.80, 0.22]	[-0.86, -0.03]	[-0.70, 0.83]	[-1.91, -0.42]	[-0.04, -0.02]	[-0.07, -0.04]
	0.043	0.265	0.036	0.861	0.002	<0.001	<0.001

Notes: The treatment-assignment models controlled for socio-demographic factors (age, age-squared, sex, marital status, living with children, living area, education, household income, employment status, and area deprivation). The outcome model additionally controlled for gap and technical covariates of cell composition.

Table S6 Estimated average treatment effect, 95% confidence interval and p value for PA frequency, diversity and activeness measures from doubly robust estimation using IPWRA (sensitivity analysis: age≥40, n=2,768)

	Hannum	Horvath2013	Horvath2018	Lin	PhenoAge	DunedinPoAm	DunedinPACE
Frequency:							
< monthly	0.30	0.35	0.21	0.22	-0.33	-0.004	-0.02
	[-0.13, 0.72]	[-0.10, 0.80]	[-0.16, 0.57]	[-0.40, 0.84]	[-0.99, 0.33]	[-0.01, 0.01]	[-0.03, 0.001]
	0.168	0.129	0.265	0.490	0.326	0.389	0.058
Monthly	0.03	-0.02	-0.19	-0.01	-0.94	-0.02	-0.04
	[-0.44, 0.50]	[-0.55, 0.51]	[-0.61, 0.23]	[-0.71, 0.70]	[-1.68, -0.21]	[-0.03, -0.01]	[-0.05, -0.02]
	0.904	0.937	0.385	0.988	0.012	<0.001	<0.001
Weekly	0.15	-0.03	-0.08	0.26	-0.78	-0.02	-0.04
	[-0.20, 0.49]	[-0.38, 0.32]	[-0.39, 0.22]	[-0.28, 0.79]	[-1.30, -0.25]	[-0.03, -0.01]	[-0.06, -0.03]
	0.401	0.879	0.594	0.344	0.004	<0.001	<0.001
Diversity:							
Low	0.21	0.06	0.11	-0.10	-0.31	-0.01	-0.02
	[-0.18, 0.61]	[-0.36, 0.48]	[-0.23, 0.45]	[-0.71, 0.51]	[-0.94, 0.32]	[-0.02, 0.002]	[-0.04, -0.01]
	0.289	0.780	0.539	0.749	0.332	0.111	0.006
Medium	0.45	0.07	0.07	0.48	-0.47	-0.02	-0.03
	[0.07, 0.84]	[-0.32, 0.45]	[-0.26, 0.40]	[-0.12, 1.08]	[-1.05, 0.11]	[-0.02, -0.01]	[-0.04, -0.02]
	0.021	0.741	0.695	0.115	0.109	<0.001	<0.001
High	0.00	-0.28	-0.17	0.02	-0.97	-0.02	-0.05
	[-0.57, 0.57]	[-0.78, 0.22]	[-0.60, 0.27]	[-0.62, 0.66]	[-1.58, -0.36]	[-0.03, -0.01]	[-0.07, -0.04]
	0.994	0.271	0.454	0.953	0.002	<0.001	<0.001
Activity							
Low	0.00	0.05	-0.20	-0.14	-0.74	-0.01	-0.005
	[-0.38, 0.38]	[-0.35, 0.46]	[-0.51, 0.11]	[-0.68, 0.41]	[-1.32, -0.16]	[-0.02, -0.003]	[-0.02, 0.01]
	0.994	0.795	0.204	0.615	0.013	0.008	0.492
Medium	-0.05	0.05	-0.15	0.42	-0.62	-0.01	-0.03
	[-0.46, 0.35]	[-0.38, 0.48]	[-0.46, 0.17]	[-0.18, 1.02]	[-1.21, -0.03]	[-0.02, -0.01]	[-0.04, -0.01]

	Hannum	Horvath2013	Horvath2018	Lin	PhenoAge	DunedinPoAm	DunedinPACE
	0.802	0.829	0.353	0.174	0.040	<0.001	<0.001
High	-0.16	-0.21	-0.29	-0.18	-1.23	-0.02	-0.03
	[-0.57, 0.25]	[-0.65, 0.23]	[-0.61, 0.04]	[-0.77, 0.41]	[-1.80, -0.65]	[-0.03, -0.01]	[-0.05, -0.02]
	0.452	0.355	0.082	0.549	<0.001	<0.001	<0.001
Very high	-0.41	-0.43	-0.57	-0.04	-1.57	-0.02	-0.05
	[-0.92, 0.10]	[-0.93, 0.06]	[-0.99, -0.15]	[-0.74, 0.65]	[-2.25, -0.90]	[-0.03, -0.02]	[-0.07, -0.04]
	0.116	0.085	0.008	0.902	<0.001	<0.001	<0.001

Notes: The treatment-assignment models controlled for socio-demographic factors (age, age-squared, sex, marital status, living with children, living area, education, household income, employment status, and area deprivation). The outcome model additionally controlled for gap and technical covariates of cell composition.
